# Lessons Learned: Quality Analysis of Optical Coherence Tomography in Neuromyelitis Optica

**DOI:** 10.1002/acn3.70235

**Published:** 2025-11-17

**Authors:** Hadi Salih, Sara Samadzadeh, Charlotte Bereuter, Seyedamirhosein Motamedi, Claudia Chien, Pablo Villoslada, Hadas Stiebel‐Kalish, Nasrin Asgari, Yang Mao‐Draayer, Marius Ringelstein, Joachim Havla, Marco Aurélio Lana Peixoto, Ho Jin Kim, Jacqueline Palace, Maria Isabel Leite, Srilakshmi M. Sharma, Fereshteh Ashtari, Rahele Kafieh, Lekha Pandit, Orhan Aktas, Philipp Albrecht, Letizia Leocani, Itay Lotan, Sasitorn Siritho, Jérôme de Seze, Romain Marignier, Caroline Froment Tilikete, Denis Bernardi Bichuetti, Ivan Maynart Tavares, Ayse Altintas, Anu Jacob, Saif Huda, Ibis Soto de Castillo, Lawrence J. Cook, Michael R. Yeaman, Axel Petzold, Alexander U. Brandt, Friedemann Paul, Frederike C. Oertel, Hanna G. Zimmermann, with the Guthy Jackson Charitable Foundation—International Clinical Consortium and CROCTINO Collaborators

**Affiliations:** ^1^ Experimental and Clinical Research Center, Max Delbrück Center for Molecular Medicine and Charité—Universitätsmedizin Berlin, Corporate Member of Freie Universität Berlin and Humboldt‐Universität zu Berlin Berlin Germany; ^2^ Neuroscience Clinical Research Center, Charité—Universitätsmedizin Berlin, Corporate Member of Freie Universität Berlin and Humboldt‐Universität zu Berlin Berlin Germany; ^3^ Institute of Regional Health Research University of Southern Denmark Odense Denmark; ^4^ Department of Neurology, The Center for Neurological Research Slagelse Hospitals Slagelse Denmark; ^5^ Department of Psychiatry and Neurosciences Charité—Universitätsmedizin Berlin, Corporate Member of Freie Universität Berlin and Humboldt‐Universität zu Berlin Berlin Germany; ^6^ Department of Neurology Hospital del Mar—Pompeu Fabra University Barcelona Spain; ^7^ Neuro‐Ophthalmology Division, Department of Ophthalmology Rabin Medical Center Tel Aviv Israel; ^8^ Eye Laboratory, Felsenstein Medical Research Center Tel Aviv University Tel Aviv Israel; ^9^ Faculty of Medicine Tel Aviv University Tel Aviv Israel; ^10^ Department of Neurology Slagelse Hospitals Slagelse Denmark; ^11^ Institute of Regional Health Research and Molecular Medicine University of Southern Denmark Odense Denmark; ^12^ Autoimmunity Center of Excellence, Multiple Sclerosis Center of Excellence, Arthritis and Clinical Immunology, Oklahoma Medical Research Foundation Oklahoma City Oklahoma USA; ^13^ Department of Neurology University Clinic and Medical Faculty, Heinrich Heine University Düsseldorf Düsseldorf Germany; ^14^ Department of Neurology Center for Neurology and Neuropsychiatry, LVR‐Klinikum, Heinrich‐Heine‐University Düsseldorf Düsseldorf Germany; ^15^ Institute of Clinical Neuroimmunology, LMU Hospital, Ludwig‐Maximilians‐University Munich Munich Germany; ^16^ CIEM MS Research Center—Federal University of Minas Gerais Belo Horizonte Brazil; ^17^ Department of Neurology National Cancer Center Goyang Republic of Korea; ^18^ Department of Neurology Oxford University Hospitals, National Health Service Trust Oxford UK; ^19^ Department of Ophthalmology Oxford University Hospitals, National Health Service Trust Oxford UK; ^20^ Kashani MS Center, Isfahan Neuroscience Research Center Isfahan University of Medical Sciences Isfahan Iran; ^21^ Department of Engineering Durham University Durham UK; ^22^ Center for Advanced Neurological Research, KS Hegde Medical Academy Nitte University Mangalore India; ^23^ Department of Neurology Kliniken Maria Hilf GmbH Mönchengladbach Mönchengladbach Germany; ^24^ Department of Medicine Los Angeles Biomedical Research Institute at Harbor‐University of California at Los Angeles (UCLA) Medical Center Torrance California USA; ^25^ IRCSS Scientific Institute San Raffaele Milan Italy; ^26^ Department of Neurorehabilitation Sciences Casa di Cura Igea Milan Italy; ^27^ Department of Neurology Rabin Medical Center, Faculty of Medicine, Tel Aviv University Tel Aviv Israel; ^28^ Division of Neurology, Department of Medicine, Siriraj Neuroimmunology Center Siriraj Hospital and Bumrungrad International Hospital Bangkok Thailand; ^29^ Neurology Service, University Hospital of Strasbourg Strasbourg France; ^30^ Neurology, Multiple Sclerosis, Myelin Disorders and Neuroinflammation, Pierre Wertheimer Neurological Hospital, Hospices Civils de Lyon Lyon France; ^31^ Department of Neuro‐Ophthalmology Hospices Civils de Lyon Lyon France; ^32^ Departamento de Neurologia e Neurocirurgia Escola Paulista de Medicina, Universidade Federal de São Paulo São Paulo Brazil; ^33^ Department of Ophthalmology and Visual Sciences Escola Paulista de Medicina, Universidade Federal de São Paulo São Paulo Brazil; ^34^ Neurology Department, School of Medicine Koc University Istanbul Turkey; ^35^ Neurology Department, Cerrahpasa School of Medicine Istanbul University Istanbul Turkey; ^36^ The Walton Centre for Neurology and Neurosurgery Liverpool UK; ^37^ Cleveland Clinic Abu Dhabi Abu Dhabi UAE; ^38^ Department of Pharmacology and Therapeutics Institute of Systems, Molecular and Integrative Biology, University of Liverpool Liverpool UK; ^39^ Department of Neurology The Walton Centre NHS Foundation Trust Liverpool UK; ^40^ Department of Neurology Hospital Clinico de Maracaibo Maracaibo Venezuela; ^41^ Department of Pediatrics University of Utah Salt Lake City Utah USA; ^42^ Department of Medicine David Geffen School of Medicine at UCLA Los Angeles California USA; ^43^ Department of Medicine, Divisions of Molecular Medicine and Infectious Diseases Harbor‐UCLA Medical Center Torrance California USA; ^44^ Department of Neuroinflammation Queen Square MS Centre, Faculty of Brain Sciences, UCL Queen Square Institute of Neurology London UK; ^45^ Moorfields Eye Hospital and The National Hospital for Neurology and Neurosurgery University College London London UK; ^46^ Department of Neurology Charité – Universitätsmedizin Berlin, Corporate Member of Freie Universität Berlin, Humboldt‐Universität zu Berlin, and Berlin Institute of Health Berlin Germany; ^47^ Einstein Center Digital Future Berlin Germany

**Keywords:** neuromyelitis optica spectrum disorders, OCT scan quality, optic neuritis, optical coherence tomography, OSCAR‐IB criteria

## Abstract

**Introduction:**

Optical coherence tomography (OCT)‐derived retina measurements are markers for neuroaxonal visual pathway status. High‐quality OCT scans are essential for reliable measurements, but their acquisition is particularly challenging in eyes with severe visual impairment, as often observed in neuromyelitis optica spectrum disorders (NMOSD).

**Objective:**

To investigate OCT quality issues in real‐world data from the international Collaborative Retrospective Study on Retinal OCT in Neuromyelitis Optica (CROCTINO).

**Methods:**

We evaluated the quality of peripapillary and macular OCT scans, using Heidelberg Spectralis SD‐OCT, Carl Zeiss Cirrus HD‐OCT, or Topcon SD‐OCT across 22 centers. Experienced graders applied OSCAR‐IB criteria for OCT quality. Eyes were classified as severely visually impaired or not based on a 1.0 logMAR cut‐off. Quality outcomes were compared using the Chi‐square test.

**Results:**

A total of 3075 OCT scans (1630 peripapillary, 1445 macular) from 539 people with NMOSD and related conditions were evaluated. Macular scans were rejected more often than peripapillary scans due to quality issues (20.1% vs. 14.5%, *p* < 0.001). Rejection rates were higher in eyes with severe visual impairment (peripapillary: 28.9%, macular: 41.6%) compared to eyes without severe visual impairment (peripapillary: 10.7%, *p* < 0.001; macular: 14.6%, *p* < 0.001).

**Conclusion:**

Our study revealed that approximately one in six scans was rejected due to low quality, with higher rejection rates in eyes with severe visual impairment. As scan quality can bias quantitative outcomes and artificial intelligence applications, these findings emphasize the unmet need for standardized OCT practices tailored to NMOSD and other conditions involving severe visual impairment.

**Abbreviations**
AMD: age‐related macular degeneration
AQP4‐IgG: aquaporin‐4 immunoglobulin G
CROCTINO: Collaborative Retrospective Study of Retinal Optical Coherence Tomography in Neuromyelitis Optica
GCIPL: ganglion cell‐inner plexiform layer
MOGAD: MOG‐IgG associated disease
MOG‐IgG: myelin oligodendrocyte glycoprotein antibody
NMOSD: neuromyelitis optica spectrum disorders
OCT: optical coherence tomography
ON: optic neuritis
ONL: outer nuclear layer
OPL: outer plexiform layer
OSCAR‐IB: criteria for OCT scan quality
pRNFL: peripapillary retinal nerve fiber layer
RNFL: retinal nerve fiber layer
SD‐OCT: spectral‐domain optical coherence tomography

## 1 Introduction

Neuromyelitis optica spectrum disorders (NMOSD) and myelin oligodendrocyte glycoprotein antibody‐associated disorders (MOGAD) are rare autoimmune diseases of the central nervous system [1, 2]. Optic neuritis (ON) is a common clinical hallmark of NMOSD and MOGAD and often results in impaired vision up to profound severe visual impairment [3, 4]. The retinal manifestations of neuroaxonal damage associated with ON can be quantified via retinal thickness measurements from optical coherence tomography (OCT) [5]. However, OCT images can be subject to various artefacts and quality issues, such as motion artefacts, poor signal strength, segmentation errors, or scan distortions caused by retinal pathologies like edema, scarring, or atrophy [6, 7]. These issues can lead to incorrect thickness measurements, analytical errors, and potentially inappropriate clinical decisions [8, 9]. Therefore, consistent and high‐quality OCT data acquisition is crucial for reliable measurements [10]. The OSCAR‐IB criteria represent a set of validated consensus criteria for retinal OCT quality control in multiple sclerosis (MS) [11, 12]. Its acronym refers to multiple parameters of OCT quality control: O = *Obvious* problems; S = poor *Signal* strength; C = *Centration* of scan; A = *Algorith*m failure; *R = Retinal Pathology* other than MS‐related; I = *Illumination*; and B = *Beam Placement*.

The Collaborative Retrospective Study of Retinal Optical Coherence Tomography in Neuromyelitis Optica (CROCTINO) collected data from 539 people with NMOSD and related disorders and brought significant insights to the forefront [13]. The main results showed that the number of ON attacks profoundly affects the extent of retinal neuroaxonal damage in individuals diagnosed with NMOSD seropositive for antibodies against aquaporin‐4 (AQP4‐IgG+) [14], MOGAD [15], and double‐seronegative NMOSD [16]. Furthermore, the study revealed that outer retinal layer thinning is not a characteristic feature of AQP4‐IgG+ NMOSD [17], highlighted differences between acute and late onset ON in NMOSD [18], and examined the utility of inter‐eye difference OCT metrics for ON diagnosis in NMOSD and MOGAD [19, 20]. All previously published studies using the CROCTINO dataset exclusively relied on scans with acceptable quality, ensuring a high standard for data analysis.

To allow for accurate quantitative analysis in CROCTINO, each OCT scan was required to meet predefined quality standards ensuring reliable measurements. The primary objective of the present study is to evaluate the systematic OCT quality control results from the CROCTINO study, conducted applying the OSCAR‐IB criteria. By quantifying the quality issues encountered in this multicenter study of NMOSD and related disorders, we aim to identify typical pitfalls and provide insights on how to address them to improve OCT data consistency and reliability in both clinical research and routine practice. This is particularly relevant for people with severe visual impairment, where OCT measurements are more challenging to obtain.

## 2 Methods

### 2.1 CROCTINO Cohort

The CROCTINO study collected OCT data from 22 centers. Retrospective OCT scans of 539 people with NMOSD and related disorders were included in the statistical analysis (Table 1). OCT devices comprise the Spectralis SD‐OCT (Heidelberg Engineering, Heidelberg, Germany), Cirrus HD‐OCT (Carl Zeiss Meditec, Dublin, CA, USA), or Topcon SD‐OCT (Topcon, Tokyo, Japan). The OCT quality control was performed in macular volume scans and peripapillary ring scans (Spectralis) or optic nerve head volume scans (Cirrus and Topcon).

**TABLE 1:** Cohort description. Adapted from Specovius et al. [13].

Participants	539
Centers	22
Age (years; mean ± SD)	43.1 ± 14.8
Sex [female, *n* (%)]	444 (82.4)
Number of peripapillary scans	1630
Number of macular scans	1445
Number of visits	1682
Participants fulfilling the 2015 diagnostic criteria for NMOSD (*n* (%))	515 (95.5)
AQP4‐IgG seropositive NMOSD [*n* (%)]	369 (68.5)
MOGAD [*n* (%)]	54 (10.0)
Double‐negative NMOSD [*n* (%)]	34 (6.3)
NMOSD with unknown antibody‐status [*n* (%)]	58 (11.0)
Participants with a history of optic neuritis [*n* (%)]	400 (74.2)
Participants with a history of myelitis [*n* (%)]	410 (76.1)

Abbreviations: AQP4‐IgG, aquaporin‐4 immunoglobulin G; MOGAD, myelin oligodendrocyte glycoprotein antibody‐associated disease; NMOSD, neuromyelitis optica spectrum disorder; SD, standard deviation.

Visual acuity was assessed using local standard procedures and was therefore not standardized across sites. A variety of chart types were used, including ETDRS and Snellen. To minimize conversion errors, visual acuity values were entered into a REDCap database via predefined dropdown options for multiple formats (decimal, logMAR, and Snellen). For analysis purposes, eyes were categorized into two clinically meaningful groups: severely impaired (visual acuity ≥ 1.0 logMAR) vs. not severely impaired.

Further details of data collection and cohort characteristics are published elsewhere [13].

In our analysis, we used a subset of scans from the protocol publication [13], selecting only the best available macular and peripapillary scan per visit for quality control, while excluding duplicates.

### 2.2 Optical Coherence Tomography Quality Control

The CROCTINO quality control was conducted during central reading at Charité—Universitätsmedizin Berlin and is based on the consensus OSCAR‐IB criteria [11]. Three experienced raters assessed the occurrence of OSCAR‐IB quality issues, with each rater evaluating a subset of the scans. Quality problems were also documented when minor quality issues occurred, yet the quality was still acceptable. The final decision regarding rejection or acceptance was based on the individual raters' discretion.

**FIGURE 1:**
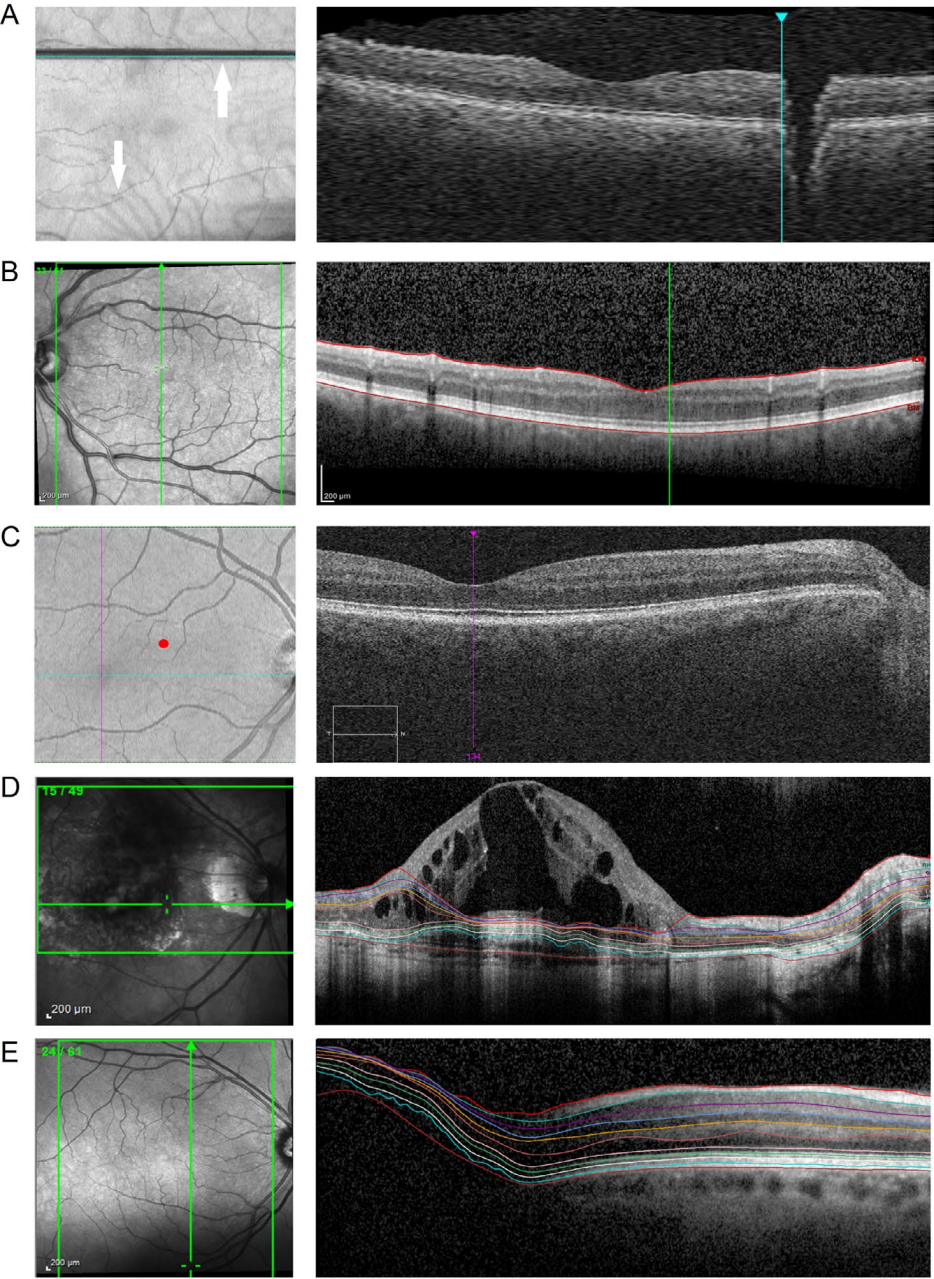
Selection of typical OCT quality problems. (A) Macular scan with *Motion* artefacts, lower arrow points at shifted B‐scans, and the upper arrow shows a blinking artefact; (B) Macular scan with B‐scan decreased *Signal* (signal strength 13 dB). (C) Macular scan with wrong *Centration*, (the red dot marks the center of the scan; the cross of the pink and the turquoise line marks the fovea, which should be the actual center of the scan). (D) Macular scan with *Retinal Pathology* classified as exudative age‐related macular degeneration, and consequently *Algorithm* failure; (E) Macular scan with insufficient *Illumination* (shadows in the lower right corner of the fundus caused by the eyelid) and, as a failed segmentation *Algorithm*, as visible in the left area of the corresponding B‐scan.

Quality categories are italicized throughout the manuscript to enhance readability. We extended and subdivided the *Obvious* criterion from the original criteria in order to categorize all artefacts and quality problems we found in our dataset. Thus, *Obvious* in this study contains the quality problems *Axial Cut‐off*, *Transversal Cut‐off*, *Motion*, *Wrong Scan Size*, *Wrong Focus*, and *Other*. In the case of *Axial Cut‐off*, a B‐scan is corrupted on the upper or lower edge, which leads to incomplete visualization of retinal layers. *Motion* problems occur due to patients' eye movements during scan acquisition (Figure 1A). They are identified via the overlay of the SLO or fundus image and the en‐face OCT image [21, 22]. *Wrong Scan Size* applies to macular scans which did not have the minimally required size of 6 mm × 6 mm, as defined in the study protocol. *Wrong Focus* issues result from insufficient correction for the eyes' refraction and manifest as blur in OCT and SLO images. OCT devices can correct for spherical refraction but not for astigmatism, leaving *Wrong Focus* issues unresolved. The second criterion, *Signal*, assesses the influence of the signal strength. Signal strength is influenced by several factors, including focus, illumination, and motion during the scan. For Spectralis, the signal strength must be higher than 15 out of 40, and for Cirrus, it should be higher than 6 out of 10 [11, 23]. For Topcon, the signal strength ranges from 0 to 100, with a minimum value of 40 required for a good quality scan [24]. However, in some assessments, when the device reports poor signal strength despite acceptable image contrast, the rater has the discretion to subjectively decide whether to accept or reject the scan (Figure 1B).

*Centration* was assessed differently in peripapillary and macular scans. In peripapillary scans, we checked whether the peripapillary ring was placed correctly on the center of the optic disc. In contrast to that, if the macular scan was not centered correctly on the fovea, we checked whether a 5 or 3 mm diameter circle area fits in the scan after centration correction (Figure 1C).

In the *Algorithm* criterion, the segmentation of the retinal layers is evaluated. While manual correction of segmentation errors is technically possible, it can be highly time‐consuming and is more practical for small datasets or minor segmentation errors. In our case, scans with major segmentation issues were rejected. For macular scans, two *Algorithm* categories were assessed: one for the total retinal thickness and one for intraretinal layers. While we used device‐internal segmentation for the peripapillary retinal nerve fiber layer (pRNFL) in peripapillary scans, we employed a device‐independent segmentation pipeline for macular volume scans [25].

The fifth criterion evaluates *Retinal Pathologies* (Figure 1D). Several studies have demonstrated that pathologies of the retina can lead to incorrect OCT measurements [26, 27, 28, 29]. A list of pathologies to consider in this context was presented by Tewarie et al. [11].

The *Illumination* criterion encompasses mainly insufficient illumination because of the beam position or vitreous haze, but also shadows from eye lashes or vitreous floaters. *Illumination* issues can cause noise and consequently affect thickness measurements (Figure 1E).

The last criterion, *Beam Placement*, refers to the positioning of the OCT light beam. *Beam placement* errors primarily affect peripapillary scans and were therefore only evaluated in those scans. The error occurs when the beam is placed outside the center of the eye [30].

The assessment of the above criteria was carefully documented in an electronic case report form (eCRF) in Research Electronic Data Capture (REDCap), a secure web application developed by Vanderbilt University for building and managing academic databases [13, 31].

A scan was not rejected by default if one or more quality issues were found, that is, if the issues were considered minor. The final decision on whether a scan was accepted or rejected was based on a rater's individual judgment. In case of rejection, the specific reason was not documented, as the cumulative effect of multiple criteria could contribute to the decision on rejection.

### 2.3 Statistical Analysis

We performed a descriptive analysis with the full dataset of scans to assess the frequencies of the quality failures within both rejected and accepted scans. We then compared the occurrence of quality failures between scans from eyes with and without severe visual impairment. To analyze the influence of visual impairment on scan quality, we created a subset of data with information on visual acuity. Within this dataset, scans were categorized as scans of eyes with severe visual impairment or scans of eyes without severe visual impairment based on their visual acuity, applying a cut‐off of ≥ 1.0 logMAR.

Categorical variables are reported as proportions (*n*, %) and continuous variables are reported as the mean ± standard deviation or median with interquartile range, depending on their distribution. Bar plots were generated to display the percentages of quality issues for rejected and accepted peripapillary and macular scans. Comparisons between different groups were performed using Pearson's Chi‐square test, with a *p*‐value threshold of < 0.05 set for determining significance.

All statistical analyses were conducted using R Studio software, version 2022.02.0 Build 443 [32]. Various libraries in R, including ggplot2 and dplyr, important for statistical analysis, were utilized for data visualization and statistical computations.

## 3 Results

Out of a total of 3075 OCT scans (1630 peripapillary scans and 1445 macular scans) evaluated, 526 (17.2%) were rejected due to quality concerns, including 236 peripapillary scans (14.5%) and 290 (20.1%) macular scans (Table 2). A Chi^2^ test (χ^2^ = 19.13, *p* < 0.001) revealed a significant difference in rejection rates, with macular scans being more frequently rejected than peripapillary scans. Notably, every evaluated scan exhibited at least one quality issue.

**TABLE 2:** Distribution of accepted and rejected OCT scans.

Decision	Peripapillary (%) *n* = 1630	Macular (%) *n* = 1445	χ^2^‐test
Accepted	1394 (85.5)	1155 (79.9)	χ^2^ = 19.13, *p* < 0.001
Rejected	236 (14.5)	290 (20.1)	

Rejection rates were higher in people with AQP4‐IgG+ NMOSD compared to MOGAD for macular, but not peripapillary scans (Tables S3 and S4). Furthermore, rejection rates were higher in ON eyes compared to non‐ON eyes in peripapillary scans (Tables S5 and S6).

### 3.1 Quality Issues in Rejected Scans

**FIGURE 2:**
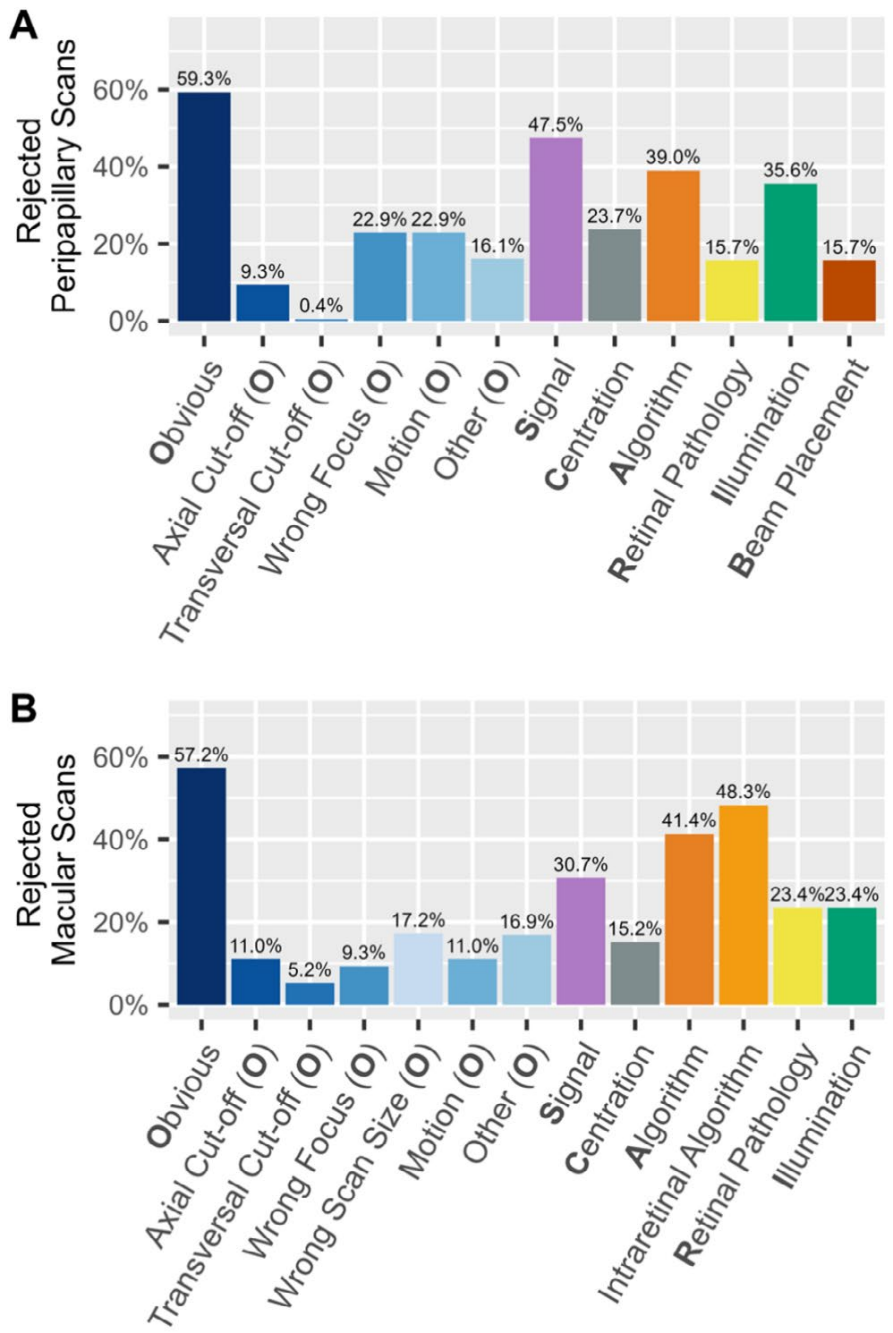
Distribution of quality issues in (A) rejected peripapillary (*n* = 236) and (B) rejected macular (*n* = 290) OCT scans.

Figure 2 illustrates a detailed breakdown of specific quality issues among rejected scans.

The most common issue in the rejected peripapillary scans was *Obvious*, which affected 59.3% of the scans. In this category, the most prevalent subcategories were *Wrong Focus* and *Motion* (both 22.9%). Further common quality issues in peripapillary scans were *Signal* (47.5%) and *Algorithm* (39.3%), indicating segmentation failures. Problems with *Illumination* and *Centration* occurred in 35.7%, and 23.7% of peripapillary scans, respectively. *Beam Placement* and *Retinal Pathology* occurred less frequently, both with 15.7% (Figure 2A).

In the rejected macular scans, the category *Obvious* was again the most prevalent, with 57.2% of the scans. *Wrong Scan Size* (17.2%) and *Motion* (11.0%) were the most common subcategories within this group. Further common quality issues in macular scans included *Intraretinal Algorithm* (48.4%), *Algorithm* (41.5%), and *Signal* (30.8%). *Illumination* and *Centration* issues occurred in 23.4% and 15.2% of the scans, respectively (Figure 2B).

When comparing the rejected macular and peripapillary scans, *Obvious* was the most common quality issue in both scan types, with similar frequency (59.3% in peripapillary scans, 57.2% in macular scans). *Signal* issues were more common in peripapillary scans (47.5% vs. 30.8%), as were *Illumination* problems (35.7% vs. 23.4%). The remaining quality issues occurred with similar frequency among peripapillary and macular scans (Figure 2).

To investigate whether specific combinations of quality issues frequently led to scan rejection, we analyzed the co‐occurrence of two or more failed criteria among rejected scans. No consistent pattern emerged, and no single combination was markedly predominant. Most frequent combinations for each scan type are listed in Table S1.

There were significantly more issues in AQP4‐IgG+ NMOSD compared to MOGAD regarding *Algorithm* and *Illumination* problems in rejected peripapillary scans. In rejected macular scans, the frequency of *Retinal Pathology* was higher in MOGAD compared to AQP4‐IgG+ NMOSD (Figure S7). Furthermore, in eyes with ON, the frequency of *Wrong Focus* and *Retinal Pathology* in rejected macular scans was higher compared to non‐ON eyes (Figure S9).

### 3.2 Quality Issues in Accepted Scans

**FIGURE 3:**
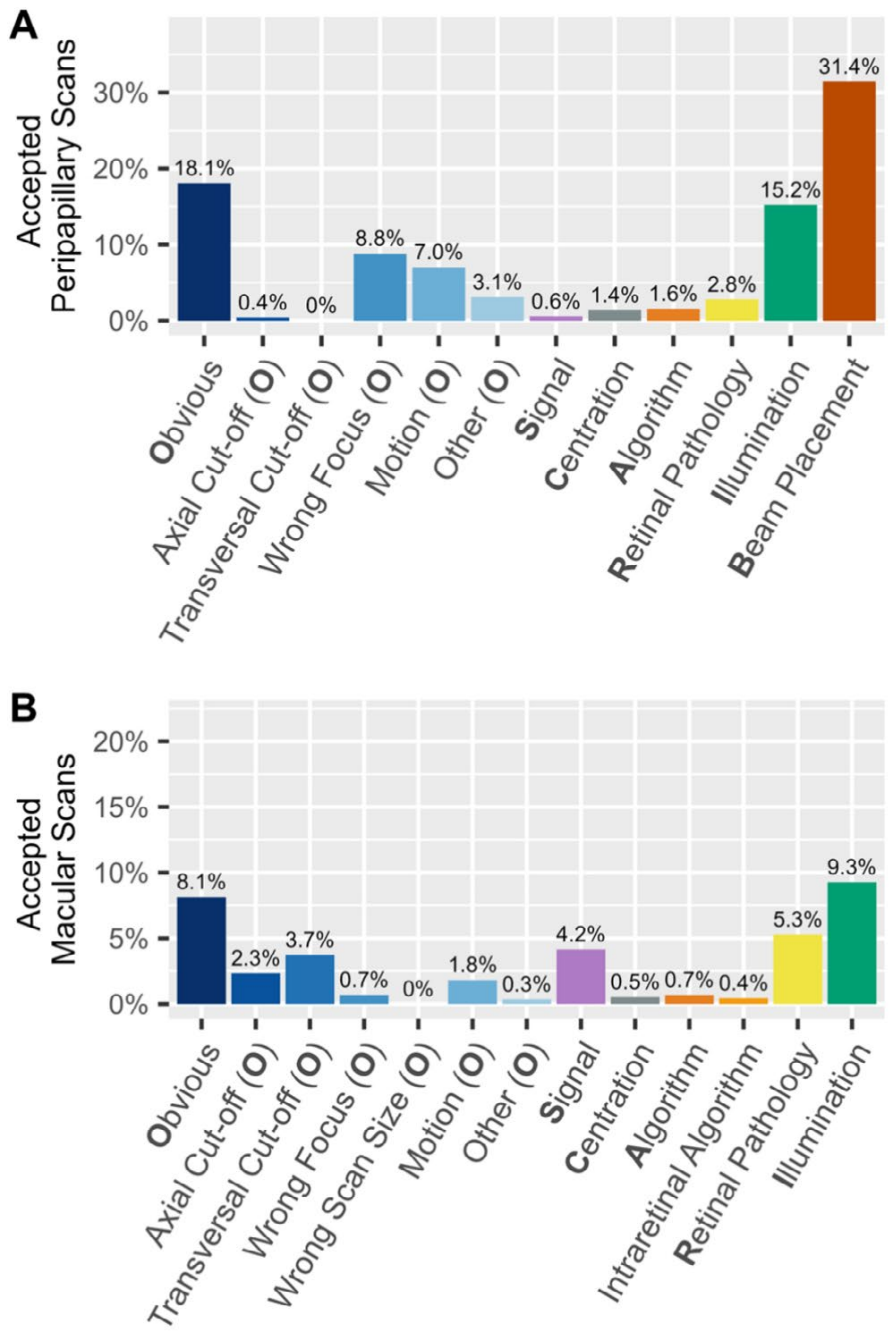
Frequency of quality issues in (A) accepted peripapillary (*n* = 1394) and (B) accepted macular scans (*n* = 1155).

The occurrence of quality issues in accepted scans is illustrated in Figure 3. The most frequently occurring quality issue in the accepted peripapillary scans was *Beam Placement*, affecting 31.4% of the scans. The second most common issue was *Obvious* (18.1%). Within this category, *Wrong Focus* (8.8%) and *Motion* (7.0%) were the most prevalent. Like *Obvious*, *Illumination* occurred with a comparable frequency (15.2%).

In accepted macular scans, *Illumination* showed the most frequent quality issue with 9.3%. Secondly, 8.1% of the scans met the *Obvious* criterion. Among its subcategories, *Transversal Cut‐off* (3.7%) and *Axial Cut‐off* (2.3%) were the most prevalent. In 5.3% of the scans, *Retinal Pathology* was detected.

When compared, both peripapillary and macular scans feature frequent *Obvious* issues in accepted scans, though with different sub‐categories. While *Illumination* problems are also present in both types of scans, they are more prevalent in peripapillary scans. However, macular scans tend to show a higher frequency in *Signal* and *Retinal Pathologies*.

Among accepted scans with two or more failed criteria, most combinations were rare and heterogeneous. However, peripapillary scans with both *Illumination* and *Beam Placement* issues occurred more frequently than any other combination (*n* = 87, 38.3% of all accepted peripapillary scans with multiple issues), suggesting that these specific issues may be more tolerated. Full details are available in Table S2.

In accepted scans, *Obvious* and *Illumination* issues occurred significantly more frequently in AQP4‐IgG+ NMOSD than in MOGAD, both for peripapillary and macular scans. Additionally, eyes with a history of ON showed a higher prevalence of *Motion* artefacts in accepted peripapillary scans (Figures S8 and S10).

### 3.3 Effect of Severe Visual Impairment on OCT Quality

**FIGURE 4:**
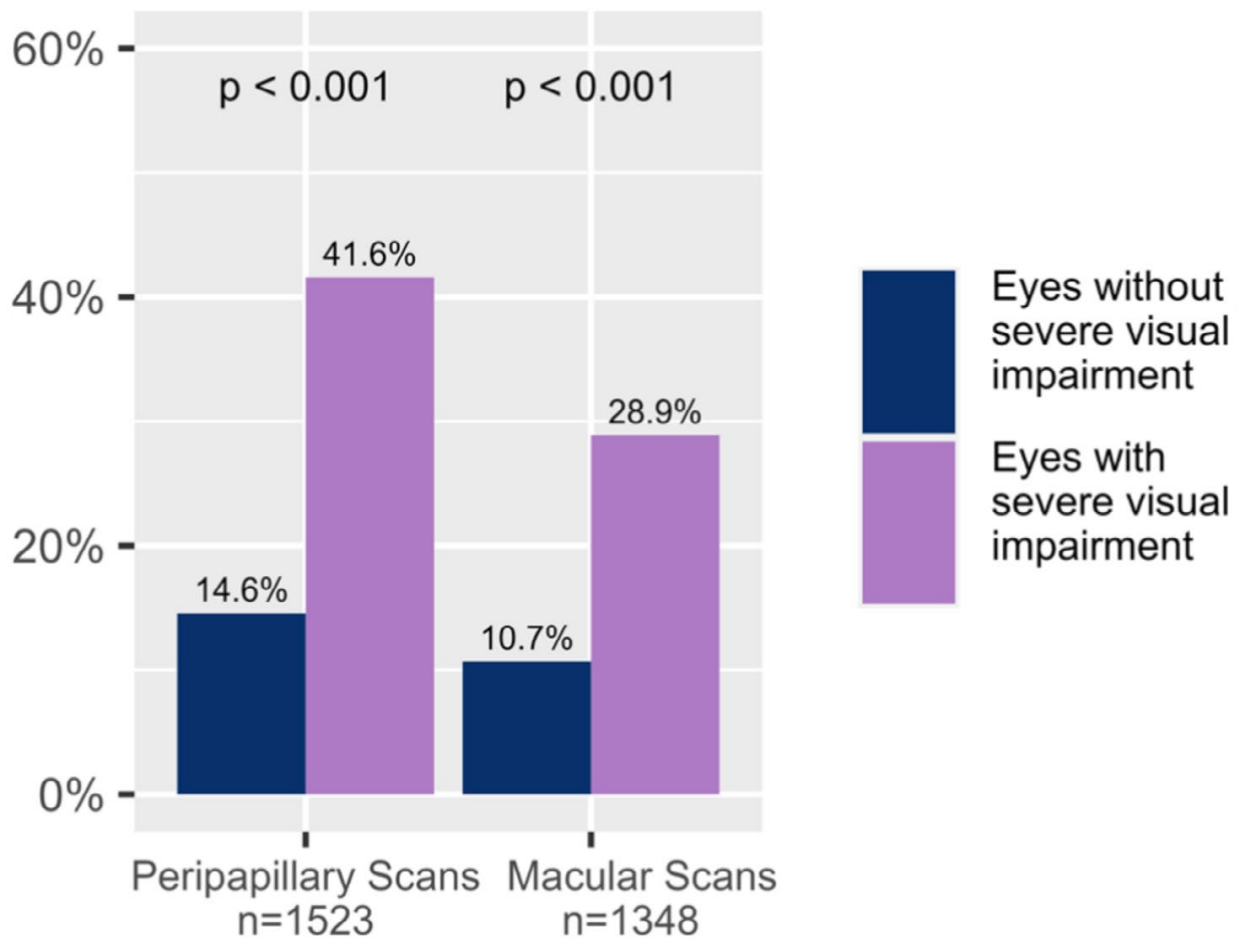
Rejection rate of OCT scans in eyes with severe visual impairment and eyes without severe visual impairment. Group comparison based on χ^2^‐test (Peripapillary Chi‐square = 60, Macular Chi‐square = 81).

A total of 2871 scans of 509 participants with available visual acuity data were analyzed to investigate the association between OCT quality and severe visual impairment, based on a ≥ 1.0 logMAR cut‐off. Within this subset, 270 of 1523 peripapillary scans (17.7%) and 202 of 1348 macular scans (15.0%) were from eyes with severe visual impairment. Figure 4 shows the rejection rates for eyes with and without severe visual impairment. Across all scan types, 162 out of 472 (34.3%) from eyes with severe visual impairment were rejected, compared to 301 out of 2399 scans (12.6%) from eyes without severe visual impairment. In both scan types, the rejection rate was higher in eyes with severe visual impairment than in eyes without severe visual impairment. In peripapillary scans, 28.9% of scans from eyes with severe visual impairment were rejected, compared to only 10.7% of scans from eyes without severe visual impairment. In the macular scans, 41.6% of scans from eyes with severe visual impairment were rejected, compared to 14.6% of scans from eyes without severe visual impairment. Differences between the groups were confirmed by Chi‐square tests (Peripapillary: χ^2^ = 60, *p* < 0.001; Macular: χ^2^ = 81, *p* < 0.001).

To investigate whether the higher rejection rate of peripapillary scans could be explained by their more frequent acquisition in severely impaired eyes, we analyzed the distribution of scan types (peripapillary vs. macular) stratified by visual acuity. In the cohort with severe visual impairment, peripapillary scans were acquired more often than macular scans (*n* = 270 vs. *n* = 202). However, this difference did not reach significance (χ^2^ = 3.72, *p* = 0.054).

**FIGURE 5:**
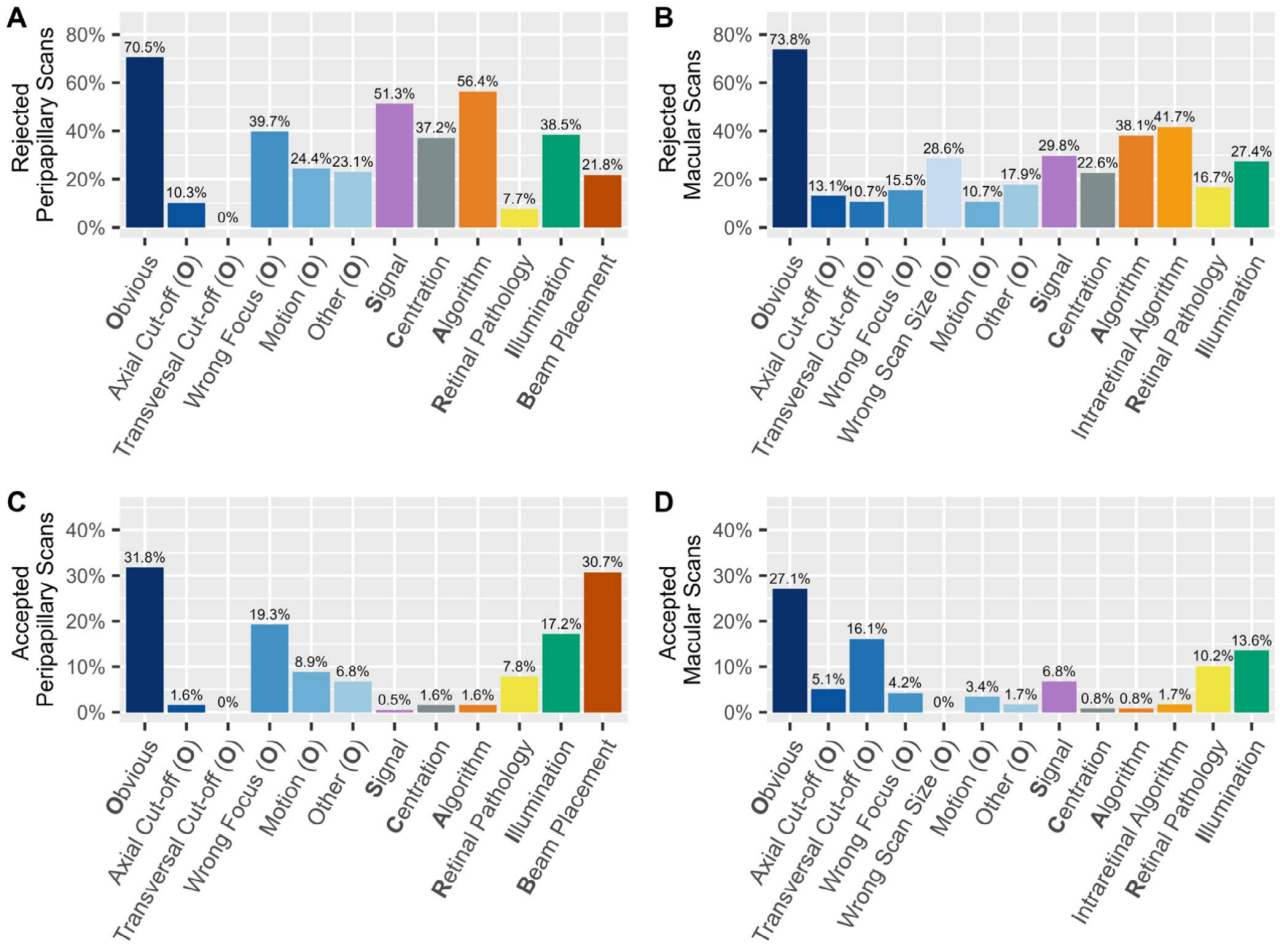
Frequency of quality issues of eyes with severe visual impairment in (A) rejected peripapillary (*n* = 78), (B) rejected macular scans (*n* = 84), (C) accepted peripapillary (*n* = 192), and (D) accepted macular scans (*n* = 118).

In rejected peripapillary scans of eyes with severe visual impairment, the most common quality criterion, *Obvious*, occurred in 70.5%. Within this category, *Wrong Focus* (39.7%), *Motion* (24.4%), and *Other* (23.1%) were the most prevalent subcategories. *Algorithm* was the second most common quality issue, occurring in 57.9% of the scans. S*ignal* problems occurred in 50% of scans. *Illumination* and *Centration* problems were found in 38.5% and 37.2% of the scans, respectively (Figure 5A).

In rejected macular scans of eyes with severe visual impairment, *Obvious* occurred in 73.8% of the scans, with *Wrong Scan Size* as the most prevalent subcategory at 28.6%. *Intraretinal Algorithm* and *Algorithm* problems occurred at 42.2% and 38.6%, respectively (Figure 5B). Across accepted scans of eyes with severe visual impairment, *Obvious* consistently remained the most frequent quality issue.

## 4 Discussion

This study investigated OCT scan quality in CROCTINO, a retrospective multicenter study of people with NMOSD and related disorders, as an example for a real‐world multi‐center dataset in people with visual impairment [13]. Our investigation highlights the prevalence of scan quality issues in OCT studies. Our main findings were: (1) one out of six scans was rejected due to quality concerns; (2) macular OCT scans were more often rejected due to quality issues than peripapillary scans, with rejection rates of 20.1% and 14.5%, respectively; (3) eyes with severe visual impairment showed a higher rejection rate and more quality issues in both macular and peripapillary scans compared to eyes without severe visual impairment (eyes with severe visual impairment: 34.3%, eyes without severe visual impairment: 12.6%); and (4) within rejected scans, the most frequent quality issues were *Obvious* with its subcategories *Wrong focus* and *Motion*, as well as *Signal* and *Algorithm*.

In this study, scans with minor quality issues were not rejected. Instead, experienced raters determined whether issues warranted rejection. Still, 17.2% of all evaluated scans had to be rejected due to quality concerns, underscoring the need for rigorous quality management in multicenter OCT studies. The higher rejection rate in peripapillary scans compared to macular scans can be attributed to the fact that most scans were obtained during clinical routine, with a larger proportion of peripapillary scans submitted to evaluate optic nerve damage caused by ON. Consequently, the increased rejection rate for peripapillary scans is likely linked to their frequent use in visually impaired patients.

To ensure high OCT scan quality in clinical studies and routine practice, it is essential to understand the factors contributing to the specific scan quality issues. In the following section, we discuss each quality criterion individually regarding its frequency and origin. Additionally, we propose targeted recommendations for future OCT protocols and operator training, derived from the literature and our own findings and experience.

*Obvious:* Peripapillary scans were more prone to *Obvious* problems than macular scans, likely due to the required nasal eye shift for acquiring the scans, which are difficult for some patients to maintain. The most common subcategories were *Motion* and *Wrong Focus*. In macular scans, the subcategory *Wrong scan size* was also common, as many clinics only run smaller sized scans or even just line scans. *Wrong Focus* was a major issue in rejected peripapillary and macular scans, whereas in accepted macular scans, cases of *Wrong Focus* were almost nonexistent. This indicates that focus issues may play a critical role in determining whether a scan can be accepted for the analysis of intraretinal layer thickness measurements. In contrast, the segmentation of the pRNFL from peripapillary scans appears more robust, allowing slightly blurred scans due to *Wrong Focus* to still be accepted. Recommendations to address this issue include ensuring optimal focus during the scanning process by improving operator training and clearer guidelines on acceptable focus levels for different scan types [33].

*Signal:* There was no notable difference in the occurrence of *Signal* issues between scans from eyes with and without severe visual impairment. This is in contrast to previous studies suggesting a connection of *Signal* issues and impaired vision [26, 34, 35, 36, 37]. The reason for this might be that signal—like all criteria—was only assessed binary on a yes/no basis. On a continuous scale, eyes with severe visual impairment might have a worse signal‐to‐noise ratio after all. Raters might consider using signal strength as a guiding measure rather than a strict binary cutoff, allowing for a certain flexibility in image quality assessment. While high signal quality is still essential, rigid cutoffs may not always be practical. For instance, if only a few B‐scans in a volume scan exhibit signal strength below the cutoff, the overall measurements may remain largely unaffected. However, in peripapillary ring scans, which consist of a single B‐Scan, lower signal strength can cause variations in retinal thickness measurements, potentially compromising the accuracy of the results [9].

*Centration* errors were notably higher in eyes with severe visual impairment. *Centration* problems are triggered mostly by patients with poor vision or fixation problems, but also depend on the operator [38]. External fixation methods or imaginary fixation points may improve outcomes for patients with severe impairment, as stated by Hansapinyo et al. and Petzold et al. [7, 39] Ensuring proper centration immediately post‐scan is crucial, especially for scans that do not allow realignment.

*Algorithm* errors showed no difference in quality between eyes with and without severe visual impairment in macular scans. However, in peripapillary scans, *Algorithm* errors occurred twice as frequently in eyes with severe visual impairment compared to eyes without severe visual impairment. This may be due to the anatomical complexity derived from structural abnormalities of eyes with severe visual impairment of NMOSD and related disorders featuring optic neuritis. The thinning of retinal layers contributes to the difficulty in accurately identifying and segmenting the different layers [27, 36, 40, 41, 42]. Furthermore, *Signal* and some of the *Obvious* errors can lead to misidentification of retinal layers, consequently resulting in *Algorithm* errors [26]. It is recommended to regularly update the OCT segmentation software, as newer versions usually provide more reliable results. While current manufacturer software is usually very reliable for a given device, several research‐based segmentation approaches have the potential to improve cross‐device robustness. Latest developments include deep learning‐based models optimized for longitudinal consistency or multi‐device harmonization [43, 44]. While not yet widely implemented in clinical practice, these tools represent an important future direction for increasing reproducibility and automation in quantitative OCT research.

*Retinal Pathology* appeared more prevalent in macular scans than in peripapillary scans. Here it is important to emphasize that the raters assessed only pathologies that are not typically associated with NMOSD. The age of the cohort indicates that many participants may have had age‐related retinal comorbidities such as age‐related macular degeneration or diabetic retinal changes, which become more visible in macular scans. When evaluating retinal pathologies, scans should not be excluded solely due to quality issues if they contain disease‐related abnormalities. These pathologies should be documented and reported, and scans with abnormalities affecting quantitative analysis should be excluded from measurements but still acknowledged in the results.

*Illumination* challenges were more prone to peripapillary scans, probably attributed to the anatomical position of the optic nerve head and difficulty avoiding iris obstruction. Partial or complete obstruction of the light beam by the iris results in suboptimal illumination, decreased signal strength, and inaccurate retinal thickness measurements [30]. A study by De Pretto et al. confirmed that partial or total blockage of the OCT beam by the iris can significantly affect illumination and signal quality. They suggest that pupil dilation should be considered in cases where *Illumination* issues are caused by small pupil size [45].

Although *Beam Placement* errors were not a major cause of scan rejection, their impact on measurement accuracy is significant. Misalignment can exceed the typical annual pRNFL loss observed in neurodegenerative diseases, emphasizing the importance of beam placement monitoring in clinical trials [30]. Interestingly, among accepted scans with multiple quality issues, the combination of *Beam Placement* and *Illumination* problems was the most common. This may indicate that these issues, although frequent, are considered less critical by raters and often tolerated when other aspects of scan quality are preserved (see Tables S1 and S2).

It is generally recommended to review OCT scans immediately after acquisition, allowing the scans to be repeated if necessary to ensure optimal quality. Poor image quality can introduce bias in artificial intelligence (AI)‐based diagnostic or prognostic models, as more severe visual impairment is often associated with lower‐quality scans. Future developments should both improve image quality and ensure that AI tools are trained and validated on datasets representative of real‐world image variability, in order to avoid such systematic bias.

Further technical advancements will be crucial for mitigating the impact of severe visual impairment on OCT scan quality in future research. Key technologies include real‐time eye tracking and motion correction, alongside post‐processing motion correction algorithms [46, 47, 48].

### 4.1 Strengths and Limitations of the Study

The strength of our study lies in the systematic quality control, which enables the individual analysis of various quality criteria, as well as the large international heterogeneous dataset of over 500 participants diagnosed with rare diseases encompassed by NMOSD. A limitation is that our quality assessment did not specify which criteria led to the rejection of a scan, as decisions often involve multiple issues. Moreover, the OSCAR‐IB criteria were originally developed to assess quality of OCT data from people with MS and have not been validated for NMOSD. Additionally, the quality control measures applied in this study reflect the characteristics of the specific study population of NMOSD and related disorders, which limits the broader applicability of our findings.

Systematic analysis of the impact of scan quality on quantitative OCT measurements was not feasible, as retinal thickness values could not be extracted due to segmentation failure or other quality issues. A further limitation is the lack of predefined thresholds for rejecting a scan. As decisions were based on expert judgment, some subjectivity in the inclusion or exclusion of scans cannot be ruled out.

Visual field data was available for a subset, but due to heterogeneity in testing protocols and missing data, it was not included in the analysis. This limited our ability to explore associations between scan rejection and visual field parameters such as mean deviation. We also did not systematically document specific types of retinal pathology observed in accepted scans, which we acknowledge as a limitation of the present analysis. Moreover, while all raters were trained in applying the OSCAR‐IB criteria, we did not formally assess interobserver variability. Evaluating interrater agreement would be valuable to quantify consistency in scan classification and further improve the reliability of quality control in multicenter studies.

## 5 Conclusion

The present findings emphasize the unmet need for rigorous, standardized quality in OCT investigations, particularly in disorders with vision loss like NMOSD.

First, scans should be reviewed immediately after acquisition to identify quality issues and, if necessary, repeated to ensure optimal scan quality. This step is critical in reducing data loss and maintaining robust datasets, particularly in challenging cases involving patients with severe visual impairment.

Second, scans should not be automatically excluded simply because they fail to fully meet quality criteria. As long as scans are fully or partially analyzable, they can still provide valuable information and should be considered for inclusion in analyses.

Thirdly, when designing studies involving people with NMOSD, it is essential to consider potential higher rejection rates for OCT scans, particularly among eyes with severe visual impairment. Our findings indicate a higher rejection rate for scans from eyes with severe visual impairment. This has direct implications for sample size calculations and recruitment strategies, which must ensure the collection of reliable quantitative data despite the likelihood of data loss.

Lastly, extensive training of OCT operators is essential to maximize good quality, though it is easier to implement in controlled trials than in resource‐limited real‐world settings. Bridging this gap is crucial for improving OCT data reliability. Standardized guidelines, like OSCAR‐IB, can serve as valuable tools for operator training and improving assessment consistency [11, 12]. This will be particularly relevant for AI‐driven analyses, which are already transitioning from research to routine care and depend on high‐quality scans.

## Author Contributions

All authors contributed to the conception of the study and the editing of the manuscript. H.S., A.U.B., F.C.O., F.P., and H.G.Z. designed the study. H.S., S.S., C.B., S.M., F.C.O., and H.G.Z. analyzed the data. All authors approved the final draft of the paper before it was submitted.

## Conflicts of Interest

C.C. has received grants from Novartis, Alexion AstraZeneca Rare Diseases; is a Standing Committee on Science Member for the Canadian Institutes of Health Research, and is part of a consortium funded by the U.S. Department of Defense; unrelated to this study. P.V. has received consultancy fees and holds stocks in Bionure Therapeutics, Accure Therapeutics, Attune Neurosciences, Adhera Health, Clight, QMENTA, Clarity, Oculis, and NeuroPrex. H.S.‐K. reports research support from Quarck, travel support, and personal fees from Quarck and Roche. Y.M.‐D. has served as a consultant and/or received grant support from: Acorda, Bayer Pharmaceutical, Biogen Idec, Celgene, EMD Serono, Genzyme, Novartis, Questor, Chugai, and Teva Neuroscience and is currently supported by grants from NIH NIAID Autoimmune Center of Excellence: UM1‐AI110557; NIH NINDS R01‐NS080821. M.R. received speaker honoraria from Novartis, Bayer Vital GmbH, Roche, Alexion, Horizon/Amgen, and Ipsen, and travel reimbursement from Bayer Schering, Biogen Idec, Merz, Genzyme, Teva, Roche, Alexion, Horizon/Amgen, and Merck, none related to this study. J.H. reports a grant for OCT research from the Friedrich‐Baur‐Stiftung, Horizon, Roche, and Merck; personal fees and nonfinancial support from Alexion, Amgen, Biogen, BMS, Horizon, J&J, Neuraxpharm, Novartis, Merck, Roche; and nonfinancial support from the Sumaira‐Foundation and Guthy‐Jackson Charitable Foundation, all outside the submitted work. M.A.L.P. has received funding for travel and speaker honoraria from Novartis, Horizon Therapeutics, and Roche. H.J.K. has received a grant from the National Research Foundation of Korea and research support from AprilBio, Eisai, Good T cells, and UCB; received consultancy/speaker fees from Alexion, Altos Biologics, AstraZeneca, Biogen, Daewoong Pharmaceutical, Eisai, GC Pharma, Handok Pharmaceutical, Kaigene, Kolon Life Science, MDimune, Merck, Mitsubishi Tanabe Pharma, Roche, and Sanofi; is a co‐editor for the Multiple Sclerosis Journal and an associate editor for the Journal of Clinical Neurology. Jacqueline Palace has received support for scientific meetings and honorariums for advisory work from Merck Serono, Novartis, Chugai, Alexion, Roche, Medimmune, Amgen, Vitaccess, UCB, Mitsubishi, Amplo, and Janssen. Grants from Alexion, Argenx, Clene, Roche, Medimmune, and Amplo biotechnology. Patent ref. P37347WO and license agreement Numares multimarker MS diagnostics. Shares in AstraZeneca. Her group has been awarded an ECTRIMS fellowship and a Sumaira Foundation grant to start later this year. A Charcot fellow worked in Oxford 2019–2021. She acknowledges partial funding to the trust by Highly Specialized Services NHS England. She is on the medical advisory boards of the Sumaira Foundation and MOG Project charities, is a member of the Guthy‐Jackson Foundation Charity, and is on the Board of the European Charcot Foundation and a member of MAGNIMS and the UK NHSE IVIG Committee and chairman of the NHSE Neuroimmunology Patient Pathway and ECTRIMS Council member on the educational committee since June 2023. Currently on the ABN advisory groups for MS and neuroinflammation and recently on the neuromuscular diseases advisory group. M.I.L. is funded by the NHS (Myasthenia and Related Disorders Service and National Specialized Commissioning Group for Neuromyelitis Optica, UK) and by the University of Oxford, UK. She has been awarded research grants from UK associations for patients with myasthenia and with muscular disorders (Myaware and Muscular Dystrophy UK, respectively) and the University of Oxford. She has received speaker honoraria or travel grants from the Guthy‐Jackson Charitable Foundation, argenx, and UCB. She serves on scientific or educational advisory boards for argenx, Amgen, and UCB. S.S.I. received funding for travel and speaker honoraria from Merck Serono (Thailand), Roche (Thailand), DKSH (Thailand), Pacific Healthcare (Thailand), Easai (Thailand), Biogen Idec, UCB (Thailand), and Novartis. O.A. has received honoraria for speaking/consultation and travel grants from Bayer Healthcare, Biogen Idec, Chugai, Novartis, Medimmune, Merck Serono, and Teva and research grants from Bayer Healthcare, Biogen Idec, Novartis, and Teva. P.A. received, with the approval of the Rector of Heinrich‐Heine University and the CEO of University of Düsseldorf Hospital, personal fees, research grants, and non‐financial support from Allergan, Biogen, Celgene, Janssen Cilag, Ipsen, Merck Serono, Merz Pharmaceuticals, Novartis, and Roche, personal fees and non‐financial support from Bayer Healthcare, Teva, and Sanofi‐Aventis/Genzyme, and grants from the German Research Foundation (DFG), all outside the submitted work. L.L. received honoraria for consulting services from Merck KGaA, Hoffmann‐La Roche Ltd. R.M. serves on the scientific advisory board for MedImmune and has received funding for travel and honoraria from Biogen, Merck Serono, Novartis, Sanofi‐Genzyme, Roche, and Teva. D.B.B. has received speaking/consulting honoraria from Bayer Health Care, Biogen Idec, Merck, Sanofi‐Genzyme, TEVA, Alexion, Horizon, and Roche and had travel expenses to scientific meetings sponsored by Bayer Health Care, Merck Serono, TEVA, and Roche. I.M.T. has received speaking/consulting honoraria from Carl Zeiss Meditech, Bausch and Lomb, and Cristalia. A.A. reports personal fees from received honoraria for giving educational presentations on multiple sclerosis, NMOSD and neuroimmunology from Novartis and Alexion. Dr. Altintas has received travel and registration coverage for attending several national and international meetings from Merck‐Serono. A.J. has received compensation for advisory board, consulting, meeting attendance, and speaking from Astra Zeneca Pharmaceuticals. S.H. is partly funded by NHS England Highly Specialized Services to run a NMO UK service. S.H. is funded by the NHS (National Specialized Commissioning Group for Neuromyelitis Optica, UK). He is also funded by an NIHR SCPRA grant. M.R.Y. is founder of NovaDigm Therapeutics Inc., ImmunoTx LLC, Tegos Therapeutics LLC, and Metacin Inc. He is principal inventor on U.S. and international patents regarding anti‐infectives, immunotherapeutics, and vaccines. He has received research funding from the National Institutes of Health (NIH), National Institute of Allergy and Infectious Diseases (NIAID), and Department of Defense (DOD), United States of America. He has received speaker or advisory honoraria from Alexion/AstraZeneca, Horizon/Amgen, and Genentech/Roche. He serves as Chair Advisor to the Guthy‐Jackson Charitable Foundation. A.U.B. is cofounder and shareholder of Motognosis GmbH and Nocturne GmbH. He is named as inventor on several patent applications regarding MS serum biomarkers, OCT image analysis and perceptive visual computing. A.U.B. is now a fulltime employee and holds stocks of Eli Lilly and Corporation. His contribution to this work is his own and does not reflect Eli Lilly and Corporation. F.P. reports research grants and speaker honoraria from Bayer, Teva, Genzyme, Merck, Novartis, MedImmune and is a member of the steering committee of the OCTIMS study (Novartis), all unrelated to this work. F.C.O. currently receives research support from the Hertie Foundation, the Deutsche Forschungsgemeinschaft (DFG), and Novartis, all unrelated to this study. She received fellowship support from the American Academy of Neurology and the National MS Society until 2023. She is a board member of the International Multiple Sclerosis Visual System (IMSVISUAL) Consortium and an editorial board member of DGNeurologie. She received speaker honoraria from UCB. H.G.Z. reports grants from Novartis, unrelated to this study. The remaining authors declare that no conflicts of interest.

## Supporting information


**Table S1:** Most frequent combinations of two or more failed quality issues in rejected peripapillary and macular scans.

**Table S2:** Most frequent combinations of two or more failed quality issues in accepted peripapillary and macular scans.

**Table S3:** Distribution of accepted and rejected peripapillary OCT scans stratified by diagnosis (AQP4‐IgG+ vs. MOGAD).

**Table S4:** Distribution of accepted and rejected macular OCT scans stratified by diagnosis (AQP4‐IgG+ vs. MOGAD).

**Table S5:** Distribution of accepted and rejected peripapillary OCT scans stratified by optic neuritis history (ON vs. non‐ON).

**Table S6:** Distribution of accepted and rejected macular OCT scans stratified by optic neuritis history (ON vs. non‐ON).

**Figure S7:** Frequency of quality issues in rejected AQP4‐IgG+ (peripapillary: *n* = 185; macular: *n* = 230) and rejected MOGAD scans (peripapillary: *n* = 21; macular: *n* = 27). Significance between groups (*p* < 0.05) is indicated by corresponding *p*‐values above the bars.

**Figure S8:** Frequency of quality issues in accepted AQP4‐IgG+ (peripapillary: *n* = 981; macular: *n* = 803) and accepted MOGAD scans (peripapillary: *n* = 171; macular: *n* = 150). Significance between groups (*p* < 0.05) is indicated by corresponding *p*‐values above the bars.

**Figure S9:** Frequency of quality issues in rejected peripapillary (ON: *n* = 156; non‐ON: *n* = 80) and macular scans (ON: *n* = 172; non‐ON: *n* = 118). Significance between groups (*p* < 0.05) is indicated by corresponding *p*‐values above the bars.

**Figure S10:** Frequency of quality issues in accepted peripapillary (ON: *n* = 779; non‐ON: *n* = 615) and macular scans (ON: *n* = 626; non‐ON: *n* = 529). Significance between groups (*p* < 0.05) is indicated by corresponding *p*‐values above the bars.

## Data Availability

The data that support the findings of this study are available on reasonable request from the corresponding author. The data are not publicly available due to privacy or ethical restrictions.
