# Correction to “Lessons Learned: Quality Analysis of Optical Coherence Tomography in Neuromyelitis Optica”

**DOI:** 10.1002/acn3.70491

**Published:** 2026-07-26

H. Salih , S. Samadzadeh , C. Bereuter , et al., “Lessons Learned: Quality Analysis of Optical Coherence Tomography in Neuromyelitis Optica,” Annals of Clinical and Translational Neurology 13, no. 3 (2026): 581–592, 10.1002/acn3.70235.41246845 PMC12968470

In Figure 4 of the above‐mentioned article, illustrating the rejection rates of peripapillary and macular scans, the labels for the two scan types were inadvertently swapped during manual labeling of the figure.

The correct version of Figure 4 is provided below.

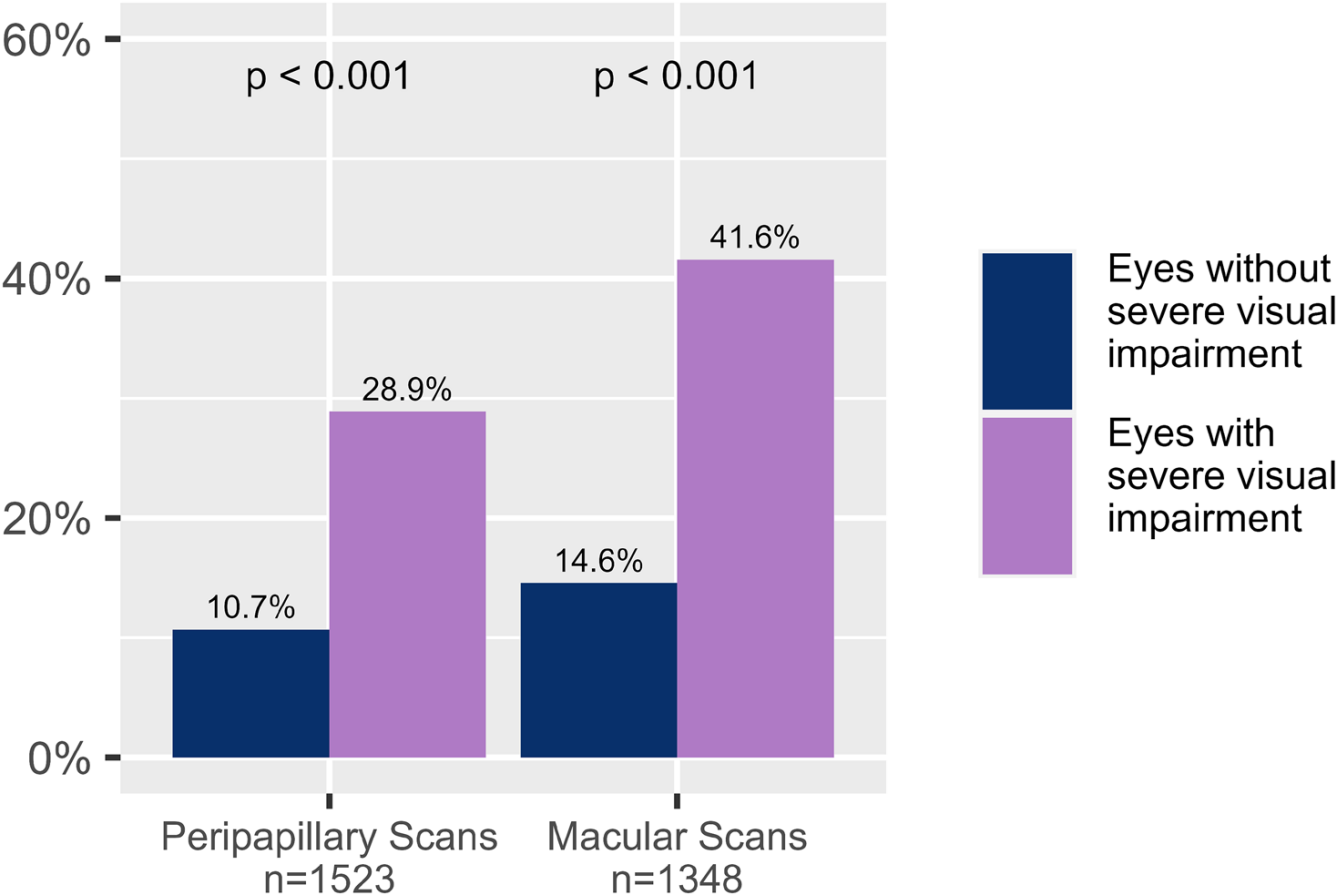

As a result, the interpretation of the rejection rates described in two paragraphs (pages 587 and 588) is incorrect. These paragraphs should be removed:

“To investigate whether the higher rejection rate of peripapillary scans could be explained by their more frequent acquisition in severely impaired eyes, we analyzed the distribution of scan types (peripapillary vs. macular) stratified by visual acuity. In the cohort with severe visual impairment, peripapillary scans were acquired more often than macular scans (*n* = 270 vs. *n* = 202). However, this difference did not reach significance (*χ* ^2^ = 3.72, *p* = 0.054).”

“The higher rejection rate in peripapillary scans compared to macular scans can be attributed to the fact that most scans were obtained during clinical routine, with a larger proportion of peripapillary scans submitted to evaluate optic nerve damage caused by ON. Consequently, the increased rejection rate for peripapillary scans is likely linked to their frequent use in visually impaired patients.”

This correction does not affect the dataset, the statistical analyses, or the main conclusions of the study.

We apologize for this error.

