# Supplementary material for: Lessons Learned: Quality Analysis of Optical Coherence Tomography in Neuromyelitis Optica
Source: Ann Clin Transl Neurol. 2025 Nov 17;13(3):581–92. doi: 10.1002/acn3.70235 (PMC12968470; doi:10.1002/acn3.70235)
Supplement: Supplementary file 1 — Table S1: Most frequent combinations of two or more failed quality issues in rejected peripapillary and macular scans. [file ACN3-13-581-s002.docx]

Supplementary Table S1: 3 Most frequent combinations of failed quality issues (≥2 criteria) in rejected peripapillary and macular scans

| Combination | n | % |
| --- | --- | --- |
| Rejected peripapillary scans (n=236) | |  |
| Scans with ≥2 criteria failed | 182 | 77.1 |
| Signal + Illumination | 12 | 6.6 |
| Motion (O) + Signal | 7 | 3.8 |
| Signal + Algorithm | 7 | 3.8 |
| Rejected macular scans (n=290) | |  |
| Scans with ≥2 criteria failed | 187 | 64.5 |
| Algorithm + Retinal Pathology | 20 | 10.7 |
| Other (O) + Algorithm | 14 | 7.5 |
| Signal + Algorithm + Illumination | 13 | 7.0 |
