# Supplementary material for: Lessons Learned: Quality Analysis of Optical Coherence Tomography in Neuromyelitis Optica
Source: Ann Clin Transl Neurol. 2025 Nov 17;13(3):581–92. doi: 10.1002/acn3.70235 (PMC12968470; doi:10.1002/acn3.70235)
Supplement: Supplementary file 2 — Table S2: Most frequent combinations of two or more failed quality issues in accepted peripapillary and macular scans. [file ACN3-13-581-s004.docx]

Supplementary Table S2: 3 Most frequent combinations of failed quality issues (≥2 criteria) in accepted peripapillary and macular scans

| Combination | n | % |
| --- | --- | --- |
| Accepted peripapillary scans (n=1394) | |  |
| Scans with ≥2 criteria failed | 377 | 16.3 |
| Illumination + Beam Placement | 87 | 38.3 |
| Retinal Pathology + Beam Placement | 18 | 7.9 |
| Motion (O) + Illumination | 16 | 7.0 |
| Accepted macular scans (n=1155) | |  |
| Scans with ≥2 criteria failed | 61 | 5.3 |
| Transversal Cut-off (O) + Illumination | 8 | 13.1 |
| Motion (O) + Illumination | 8 | 13.1 |
| Signal + Illumination | 8 | 13.1 |
