# Supplementary material for: Lessons Learned: Quality Analysis of Optical Coherence Tomography in Neuromyelitis Optica
Source: Ann Clin Transl Neurol. 2025 Nov 17;13(3):581–92. doi: 10.1002/acn3.70235 (PMC12968470; doi:10.1002/acn3.70235)
Supplement: Supplementary file 3 — Table S3: Distribution of accepted and rejected peripapillary OCT scans stratified by diagnosis (AQP4‐IgG+ vs. MOGAD). [file ACN3-13-581-s007.docx]

Supplementary Table S3: Distribution of accepted and rejected peripapillary OCT scans stratified by diagnosis (AQP4-IgG+ vs. MOGAD)

| Diagnosis | Accepted (n, %) | Rejected (n, %) | Total (n) | Chi²-Test |
| --- | --- | --- | --- | --- |
| AQP4-IgG | 981 (84.1%) | 185 (15.9%) | 1166 | χ² = 2.75,  *p* = 0.098 |
| MOGAD | 171 (89.1%) | 21 (10.9%) | 192 |  |
