# Supplementary material for: Lessons Learned: Quality Analysis of Optical Coherence Tomography in Neuromyelitis Optica
Source: Ann Clin Transl Neurol. 2025 Nov 17;13(3):581–92. doi: 10.1002/acn3.70235 (PMC12968470; doi:10.1002/acn3.70235)
Supplement: Supplementary file 4 — Table S4: Distribution of accepted and rejected macular OCT scans stratified by diagnosis (AQP4‐IgG+ vs. MOGAD). [file ACN3-13-581-s006.docx]

Supplementary Table S4: Distribution of accepted and rejected macular OCT scans stratified by diagnosis (AQP4-IgG+ vs. MOGAD)

| Diagnosis | Accepted (n, %) | Rejected (n, %) | Total (n) | Chi²-Test |
| --- | --- | --- | --- | --- |
| AQP4-IgG | 803 (77.7%) | 230 (22.3%) | 1033 | χ² = 4.01,  *p* = 0.045 |
| MOGAD | 150 (84.7%) | 27 (15.3%) | 177 |  |
