# Supplementary material for: Lessons Learned: Quality Analysis of Optical Coherence Tomography in Neuromyelitis Optica
Source: Ann Clin Transl Neurol. 2025 Nov 17;13(3):581–92. doi: 10.1002/acn3.70235 (PMC12968470; doi:10.1002/acn3.70235)
Supplement: Supplementary file 5 — Table S5: Distribution of accepted and rejected peripapillary OCT scans stratified by optic neuritis history (ON vs. non‐ON). [file ACN3-13-581-s009.docx]

Supplementary Table S5: Distribution of accepted and rejected peripapillary OCT scans stratified by optic neuritis history (ON vs. non-ON)

| Diagnosis | Accepted (n, %) | Rejected (n, %) | Total (n) | Chi²-Test |
| --- | --- | --- | --- | --- |
| Non-ON | 615 (88.5%) | 80 (11.5%) | 695 | χ² = 8.21,  *p* = 0.004 |
| ON | 779 (83.3%) | 156 (16.7%) | 935 |  |
