# Supplementary material for: Lessons Learned: Quality Analysis of Optical Coherence Tomography in Neuromyelitis Optica
Source: Ann Clin Transl Neurol. 2025 Nov 17;13(3):581–92. doi: 10.1002/acn3.70235 (PMC12968470; doi:10.1002/acn3.70235)
Supplement: Supplementary file 6 — Table S6: Distribution of accepted and rejected macular OCT scans stratified by optic neuritis history (ON vs. non‐ON). [file ACN3-13-581-s010.docx]

Supplementary Table S6: Distribution of accepted and rejected macular OCT scans stratified by optic neuritis history (ON vs. non-ON)

| Diagnosis | Accepted (n, %) | Rejected (n, %) | Total (n) | Chi²-Test |
| --- | --- | --- | --- | --- |
| Non-ON | 529 (81.8%) | 118 (18.2%) | 647 | χ² = 2.25,  *p* = 0.134 |
| ON | 626 (78.4%) | 172 (21.6%) | 798 |  |
