# Supplementary figures and images for: Lessons Learned: Quality Analysis of Optical Coherence Tomography in Neuromyelitis Optica
Source: Ann Clin Transl Neurol. 2025 Nov 17;13(3):581–92. doi: 10.1002/acn3.70235 (PMC12968470; doi:10.1002/acn3.70235)

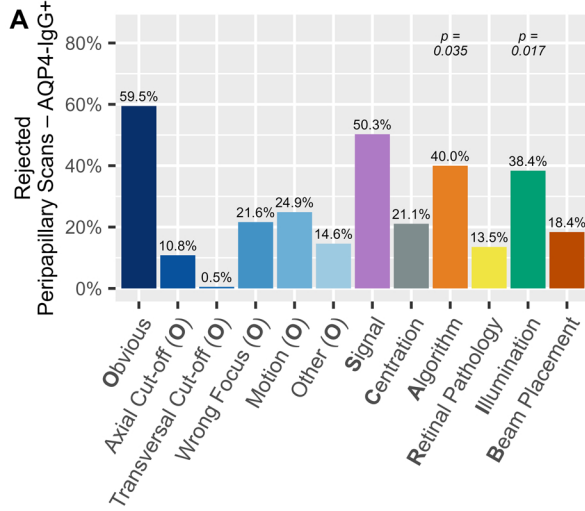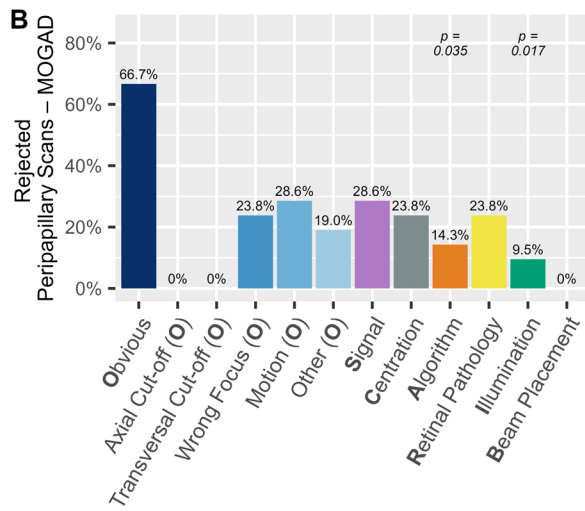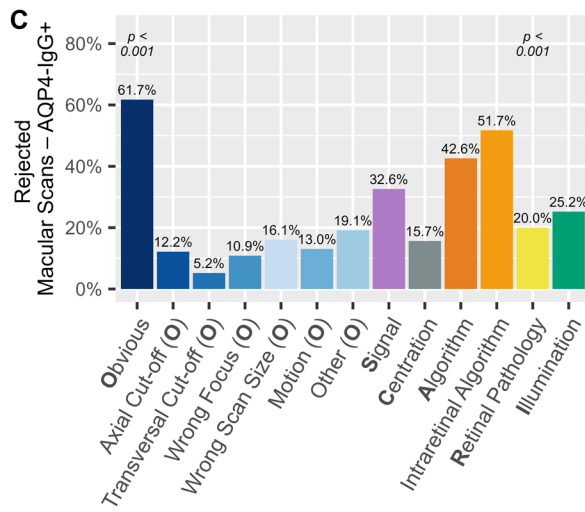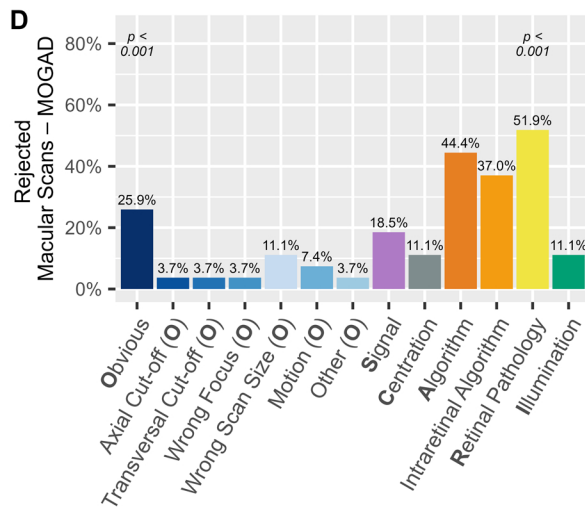

Supplement: Supplementary file 7 — Figure S7: Frequency of quality issues in rejected AQP4‐IgG+ (peripapillary: n = 185; macular: n = 230) and rejected MOGAD scans (peripapillary: n = 21; macular: n = 27). Significance between groups (p < 0.05) is indicated by corresponding p‐values above the bars. [file ACN3-13-581-s008.pdf]

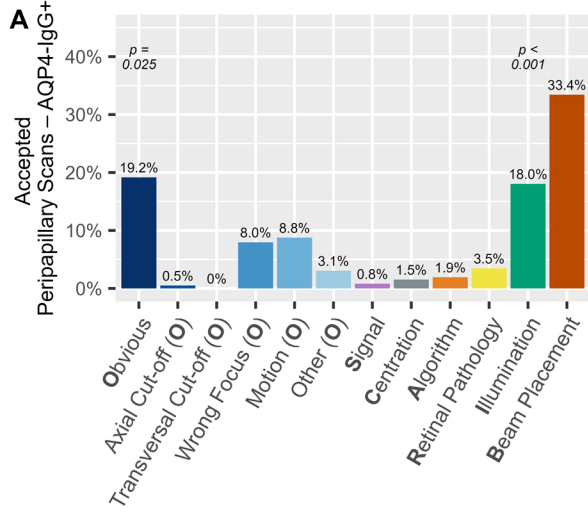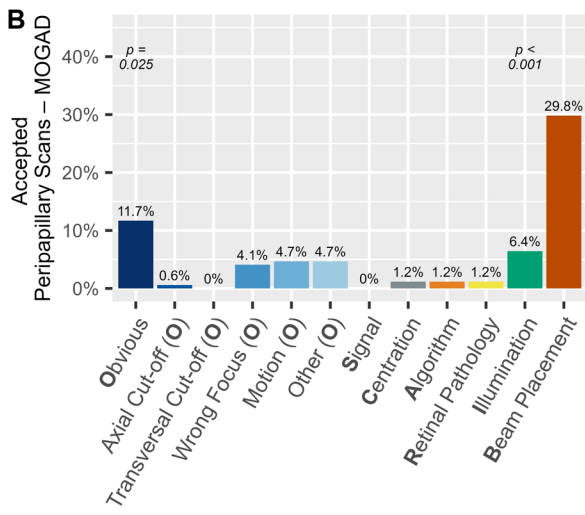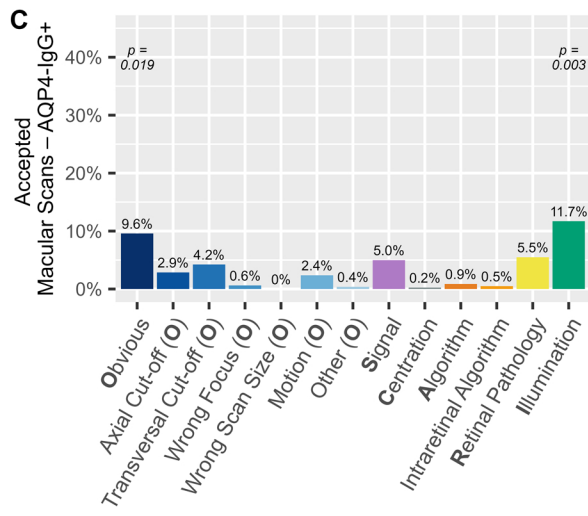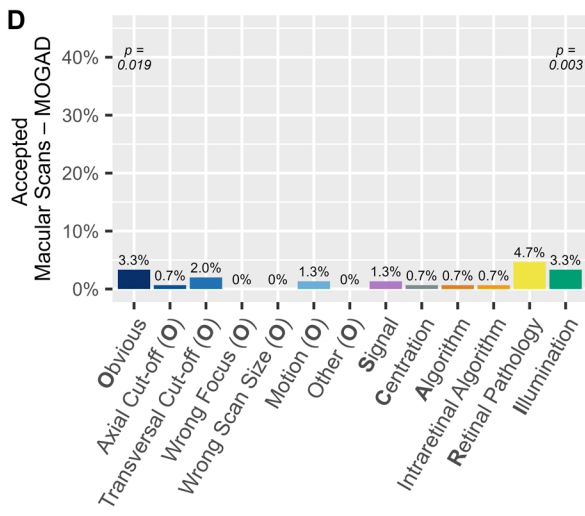

Supplement: Supplementary file 8 — Figure S8: Frequency of quality issues in accepted AQP4‐IgG+ (peripapillary: n = 981; macular: n = 803) and accepted MOGAD scans (peripapillary: n = 171; macular: n = 150). Significance between groups (p < 0.05) is indicated by corresponding p‐values above the bars. [file ACN3-13-581-s005.pdf]

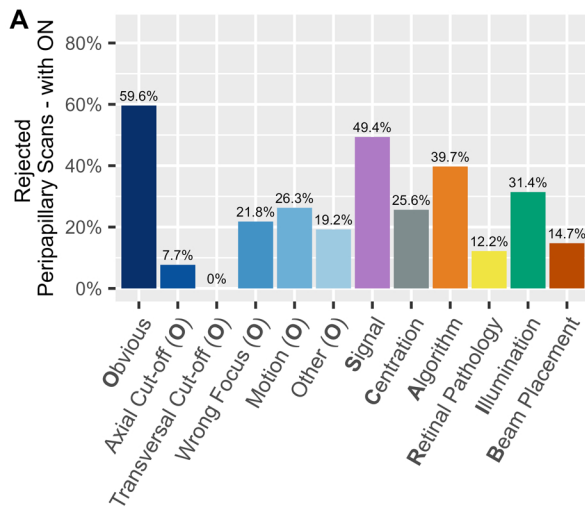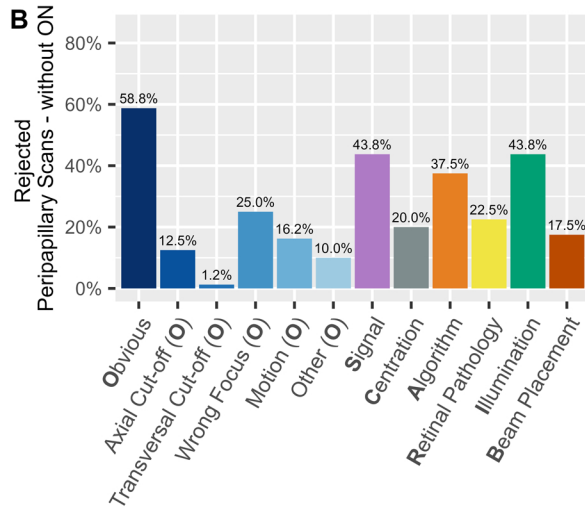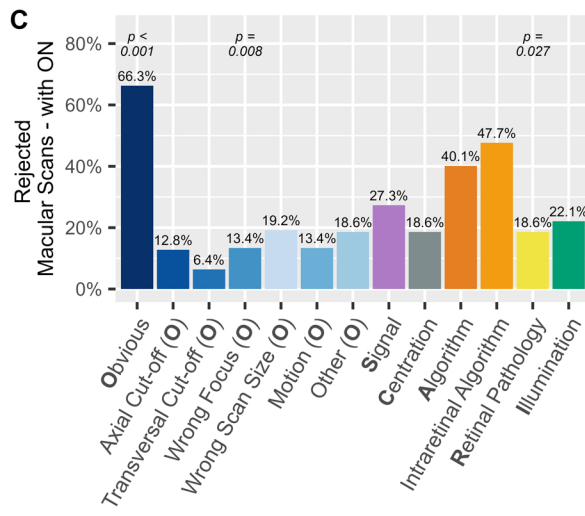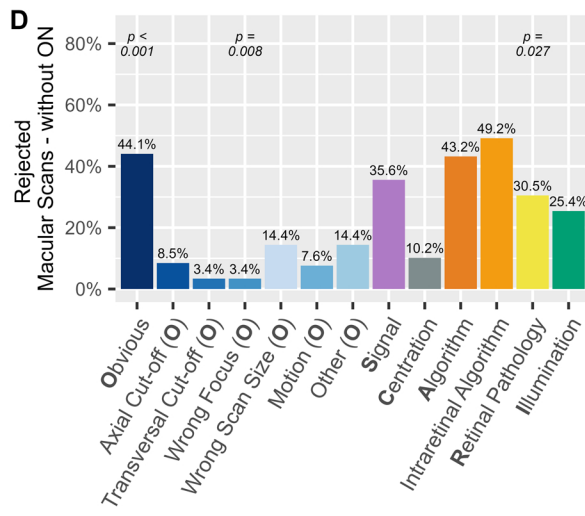

Supplement: Supplementary file 9 — Figure S9: Frequency of quality issues in rejected peripapillary (ON: n = 156; non‐ON: n = 80) and macular scans (ON: n = 172; non‐ON: n = 118). Significance between groups (p < 0.05) is indicated by corresponding p‐values above the bars. [file ACN3-13-581-s003.pdf]

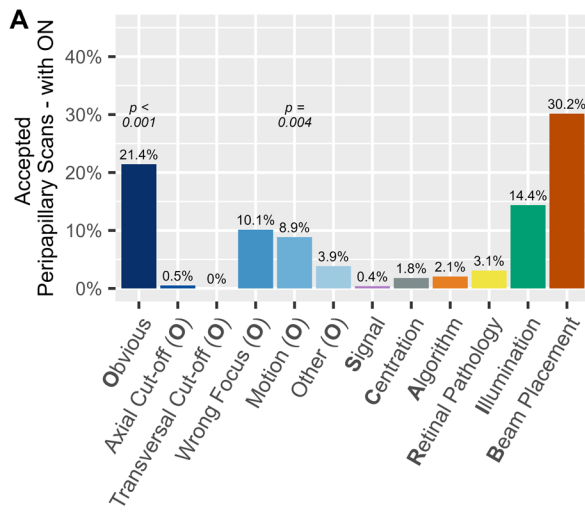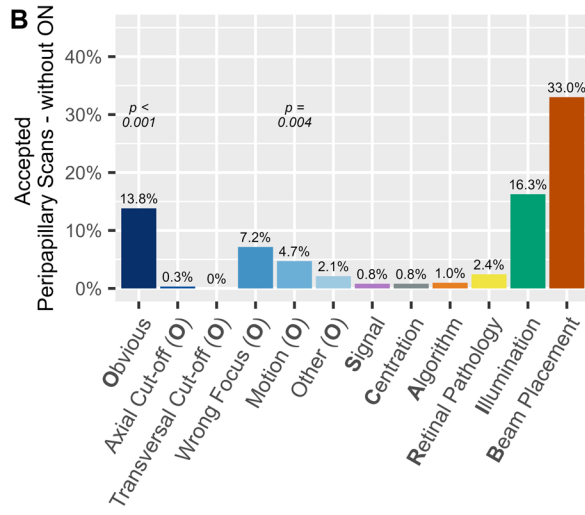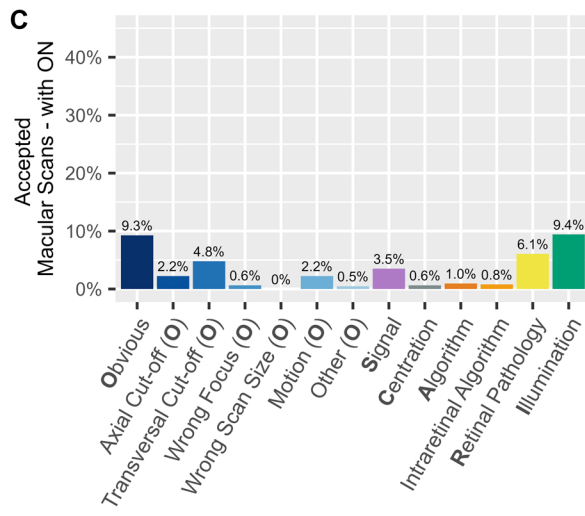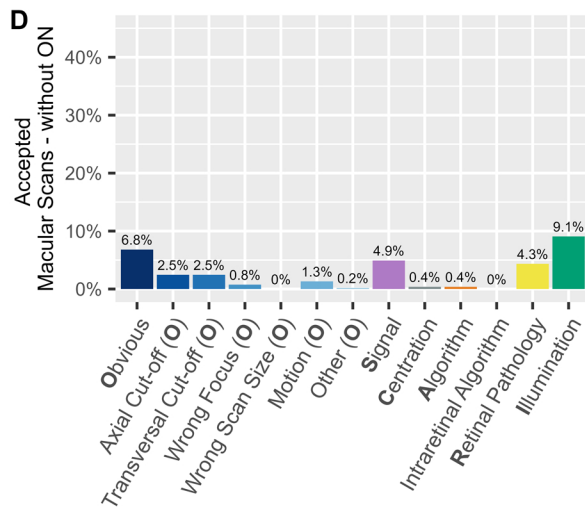

Supplement: Supplementary file 10 — Figure S10: Frequency of quality issues in accepted peripapillary (ON: n = 779; non‐ON: n = 615) and macular scans (ON: n = 626; non‐ON: n = 529). Significance between groups (p < 0.05) is indicated by corresponding p‐values above the bars. [file ACN3-13-581-s001.pdf]
